# Use of Autoreactive Antibodies in Blood of Patients with Pancreatic Intraductal Papillary Mucinous Neoplasms (IPMN) for Grade Distinction and Detection of Malignancy

**DOI:** 10.3390/cancers14153562

**Published:** 2022-07-22

**Authors:** Niall Brindl, Henning Boekhoff, Andrea S. Bauer, Matthias M. Gaida, Hien T. Dang, Jörg Kaiser, Jörg D. Hoheisel, Klaus Felix

**Affiliations:** 1 Department of General, Visceral and Transplantation Surgery, Heidelberg University Hospital, 69120 Heidelberg, Germany; joerg.kaiser@med.uni-heidelberg.de; 2 Division of Functional Genome Analysis, German Cancer Research Center (DKFZ), 69120 Heidelberg, Germany; h.boekhoff@dkfz-heidelberg.de (H.B.); andrea.bauer@dkfz-heidelberg.de (A.S.B.); j.hoheisel@dkfz-heidelberg.de (J.D.H.); 3 Institute of Pathology, University Medical Center of the Johannes Gutenberg-University Mainz, 55131 Mainz, Germany; matthias.gaida@unimedizin-mainz.de; 4 Research Center for Immunotherapy, University Medical Center of the Johannes Gutenberg-University Mainz, 55131 Mainz, Germany; 5 TRON, Translational Oncology at the University Medical Center of the Johannes Gutenberg-University Mainz, 55131 Mainz, Germany; 6 Department of Surgery, Thomas Jefferson University, Philadelphia, PA 19144, USA; hien.dang@jefferson.edu

**Keywords:** IPMN, pancreatic cancer, antibodies, protein microarray, biomarker

## Abstract

**Simple Summary:**

Risk stratification via biomarkers used with imaging could support predicting high-risk pancreatic intraductal papillary mucinous neoplasms (IPMN) with malignant potential. We explored the presence of autoreactive antibodies in the blood of patients with IPMN of different grades of dysplasia, including IPMN with associated invasive carcinoma and early-stage pancreatic ductal adenocarcinoma (PDAC), to identify signatures of early malignancy. Multivariate predictive models retained 14 proteins as potential biomarkers for discrimination between all disease classes. The integration of the autoreactive-antibody panel with clinical variables may aid in risk stratification of high-risk IPMN patients, which would subsequently improve clinical management.

**Abstract:**

(1) Background: A reliable non-invasive distinction between low- and high-risk pancreatic intraductal papillary mucinous neoplasms (IPMN) is needed to effectively detect IPMN with malignant potential. This would improve preventative care and reduce the risk of developing pancreatic cancer and overtreatment. The present study aimed at exploring the presence of autoreactive antibodies in the blood of patients with IPMN of various grades of dysplasia. (2) Methods: A single-center cohort was studied composed of 378 serum samples from patients with low-grade IPMN (*n* = 91), high-grade IPMN (*n* = 66), IPMN with associated invasive cancer (*n* = 30), pancreatic ductal adenocarcinoma (PDAC) stages T1 (*n* = 24) and T2 (*n* = 113), and healthy controls (*n* = 54). A 249 full-length recombinant human protein microarray was used for profiling the serum samples. (3) Results: 14 proteins were identified as potential biomarkers for grade distinction in IPMN, yielding high specificity but mediocre sensitivity. (4) Conclusions: The identified autoantibodies are potential biomarkers that may assist in the detection of malignancy in IPMN patients.

## 1. Introduction

Intraductal papillary mucinous neoplasms (IPMN) are primarily benign cystic tumors of the pancreas. Over time, they may progress from low-grade dysplasia to high-grade dysplasia and subsequently to invasive carcinoma [1]. IPMN show broad heterogeneity regarding their localization, type of histology, and malignant potential. They can be located within the main pancreatic duct, in side branches, or in combined forms [2]. Different grades of dysplasia and histological subtypes of IPMN are defined by pathological analysis. IPMN are categorized into lesions with low-grade dysplasia, high-grade dysplasia, or IPMN with associated invasive carcinoma [2]. Additionally, IPMN can be classified by their histological subtype, namely as intestinal, pancreaticobiliary, or gastric IPMN [3,4,5]. The extent of IPMN and their associated dysplasia are heterogenous and can affect the whole pancreas [6]. Moreover, IPMN are a risk factor for the development of pancreatic ductal adenocarcinoma (PDAC) in the area of the cystic lesion, which is then defined as PDAC derived from IPMN. In some cases, PDAC and IPMN can both be found co-existing within the same pancreas in different areas, which is then referred to as PDAC with concomitant IPMN [7].

Despite the increasing incidence of IPMN, adequate treatment of these lesions is still subject to discussion. Various guidelines were set over the last decade [4,8,9,10]. In 2018, the first evidence-based guideline was published by the European Study Group on Cystic Tumours of the Pancreas [2]. Although surgical intervention remains the only definite treatment, timing remains an issue [11]. Late intervention can lead to the evolvement of PDAC and a poor prognosis [12]. Surgery in the early stages of disease can cause unnecessary side effects and complications, leading to increased morbidity. Currently, the indication for surgery depends on various radiological and clinical findings, summarized as high-risk stigmata and worrisome features [2,4]. However, pathological findings in resected IPMN often show various grades of malignancy that are not observed during preoperative radiologic imaging (MRI/CT) [13]. Therefore, additional methods are needed for preoperative and non-invasive classification to accurately diagnose IPMN grades.

Although cancer cells and the tumor microenvironment exhibit signaling pathways to evade the immune system, tumor-associated antibodies are nevertheless already present at early-stage disease and circulate in the blood [14]. Antibodies serve as a multiplier, yielding more molecules compared with the corresponding antigens, as they are produced by the immune system, even if the tumor is still small [15]. This makes them appealing as potential biomarkers for diagnostic and prognostic purposes. The possible utilization of autoantibodies as biomarkers for cancer has already been studied for several tumor entities [16,17,18]. In this study, autoantibodies (AAbs) were detected and analyzed by protein microarray analyses [19,20,21], with the following objectives: (1) The identification of distinct antibody signatures for different pathological stages of IPMN; and (2) the assessment of the performance of AAbs signatures for the discrimination of low- and high-risk IPMN.

## 2. Materials and Methods

### 2.1. Patients and Samples

Serum samples from patients with pathologically confirmed IPMN and PDAC as well as from healthy donors were obtained from the Pancobank of the European Pancreas Center (EPZ) at the Department of Surgery, University Hospital Heidelberg. Pancobank is a member of the Biomaterial Bank Heidelberg. Ethical approval was obtained from the Ethics Committee of Heidelberg University: 301/2001 and 159/2002. Written informed consent had been obtained from all blood donors. All patients with IPMN underwent pancreatic surgery because of either the suspicion of malignancy or other indications for surgery. In this study, only sera were included for which a final pathological diagnosis confirmed either IPMN of a specific grade of dysplasia or PDAC.

### 2.2. Gene Selection and DNA Extraction

To select protein antigens of high interest, a PubMed literature search was performed. The proteins included in the study matched one or more of the following criteria: reported in previous IPMN-related antibody reports [22], pancreatic cancer-related antibody reports [18,20,23,24,25,26,27], or cancer autoantibody publications [14,28,29,30]. Additionally, some proteins of importance to pancreatic function, such as CCKBR, PNLIP, LDH, VEGF, LOX, G6PD, and GPX-4, were included. In total, 364 candidates of interest were identified. cDNAs were obtained and extracted from the hORFeome 8.1 cDNA library, which consists of 16.172 cDNAs of human genes stored in vectors in *E. coli* bacteria [31]. The library was provided by the Genomic and Proteomic Core Facility of the German Cancer Research Center (DKFZ). Because not all selected genes were available in the hORFeome 8.1 library, and some amplified PCR products did not match the quality criteria, 249 cDNAs were included in the study (Table S1). For DNA extraction, bacterial clones were grown in 96-well plates overnight and subsequently centrifuged at 2750× *g* at room temperature for 30 min. The bacterial pellets were resuspended in 100 µL ddH_2_O and heated to 75 °C for 20 min. After a second centrifugation, the supernatants containing the DNA were transferred into new plates and either immediately processed or stored at −20 °C.

### 2.3. Microarray Screening

Protein microarrays were produced and incubated as described in full detail elsewhere [20,21,32,33]. Briefly, the cDNAs were used for a PCR-based generation of protein expression templates. During PCR amplification, the functional sequences required for subsequent in vitro transcription and translation were added at either end of the construct. Moreover, a 6× His- and a V5-tag were included for quality control. All PCR products were spotted onto epoxysilane-coated slides using the non-contact Nanoplotter 2 (GeSIM, Radeberg, Germany). In a second run, each cDNA spot was covered with 8 droplets of the S30T7 high-yield cell-free protein expression system (Promega, Mannheim, Germany) and incubated under humid conditions in a ventilated incubator. Positive (Epstein-Barr Virus VCA p18) and negative controls (PCR mixture without DNA template) were also printed onto each microarray in several replicates. Success of spotting and protein expression was assessed using fluorescence-conjugated antibodies directed against 6× His- and V5-tags present at either end of each expressed protein.

Prior to incubation with blood serum, microarray slides were blocked for 1 h by orbital shaking in blocking buffer (1× PBS-T with 4% skim milk; Gerbu Biotechnik, Heidelberg, Germany), followed by three washing steps with 1× PBS-T for 5 min each. The presence of autoantibodies was assessed by incubation with a mixture of 12 µL patient serum with 333 µL blocking buffer and 55 µL *E. coli* lysate (1 mg/mL) for 2 h at 4 °C, followed by three washing steps with 1× PBS-T. A fluorescence-conjugated secondary antibody (goat anti-human IgG + IgM + IgA (H + L); Dianova GmbH, Hamburg, Germany) was diluted 1:333 in blocking buffer, added, and incubated for 1 h. Subsequently, the slides were washed three times for 7 min, rinsed in water, and air-dried in a ventilated oven at 30 °C for 20 min. Image acquisition was carried out on a Tecan PowerScanner (Tecan, Männedorf, Switzerland) at λ = 325 nm.

### 2.4. Data Analysis

GenePix Pro 6.0 software (Molecular Devices, Sunnyvale, CA, USA) was used to analyze the fluorescence intensity of each microarray spot and to eliminate spatial artifacts. Signal intensities were expressed as median fluorescence intensity (MFI). Data analysis was performed with the software R v. 4.1.1. Normalization was performed based on the negative control MFIs. Normalization was performed for each patient individually because of high variety in background due to the microarray production processes and the individual characteristics of the serum samples. In particular, distance weighted averaging was used [34]. The protein was immunoreactive if its MFI value was at least five standard deviations above the regional average, and a fold-change is computed by dividing the observed MFI by such threshold.

Approximate overlap between the age distributions of the disease classifications considered for biomarker selection was achieved, and sample weights inversely proportional to the disease class sizes were computed. A weighted multinomial lasso regression with unpenalized age and sex was fitted with the glmnet R package [35] to select a smaller subset of biomarkers that discriminate the disease categories. As one patient could be predicted to belong to one of several classes, generalizations of two-class measures to assess discriminative power were adopted. Following Hand and Till [36], pairwise areas under the curve (AUCs), as well as the global M-value, were computed. The M-value provides a global AUC for multiclass classification problems and is obtained as an average of the pairwise AUCs. Computation of the latter was performed based on an adaptation of the HandTill2001 R package [37]. Plots were produced with the ggplot2 [38] and the pheatmap [39] R packages. Biomarkers selected by the multinomial lasso were screened for their sensitivity and specificity in predicting different entities.

### 2.5. Cross-Validation

Apparent measures were computed based on the predicted disease classification obtained from the multinomial lasso and baseline models fitted on all available patient samples. As the same samples are used for both model fitting and prediction, apparent measures tend to be over-optimistic when generalizing to new patients. To counterbalance this, an outer 10-fold cross-validation procedure was adopted. Here, a subset of patients was in turn excluded from model fitting, and their disease classification was predicted. Each fold excluded approximately 10% of the total number of patients of each class. The cross-validated M-values and confusion matrices were then obtained.

### 2.6. Functional Annotation Clustering

Functional annotation clustering was performed using the Database for Annotation, Visualization, and Integrated Discovery (DAVID) [40,41] to classify identified proteins and their functionality. This included gene ontology (GO enrichment analysis [42,43]) and pathways (KEGG pathway enrichment analysis [44,45,46]).

## 3. Results

In this study, perioperatively collected sera from 324 patients were analyzed, which were grouped into two subsets: IPMN and PDAC. As a reference, 54 samples obtained from healthy individuals (Co) were tested. The IPMN group consisted of 187 samples from patients with different pathological stages, including 91 IPMN low-grade dysplasia (IPMN-LG), 66 high-grade dysplasia (IPMN-HG), and 30 IPMN with associated invasive cancer (IPMN-CA), including colloidal carcinoma and adenocarcinoma. Additionally, 137 samples from PDAC patients were included, which consisted of 24 patients with stage T1 and 113 with stage T2. Thirteen of the resected Thirteen of the resected PDAC were pathologically classified as PDAC derived from IPMN, two as PDAC with concomitant IPMN. As the primary diagnosis in these cases was PDAC, they were allocated to the PDAC cohort. Only material from patients with a pathologically confirmed diagnosis was included. Characteristics of cases and controls, including age, gender, TNM classification, tumor location, histopathological grading, and UICC stage of resection status, are shown in Table 1. Further patient information, such as comorbidities, paraneoplastic syndromes, or BMI, was only partially available. An analysis of the data showed no relevant difference between patient groups.

Out of 364 protein candidates of interest, 281 were available in the hORFeome 8.1 cDNA library [31]. An additional 32 candidates could not be amplified by PCR, which resulted in a final 249 cDNAs microarray platform (Table S1); all proteins were present as full-length molecules.

**Figure 1:**
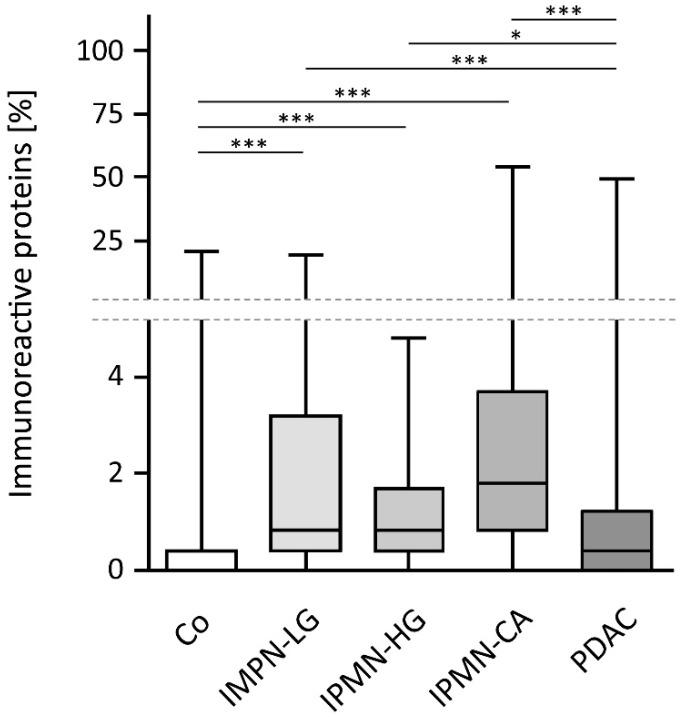
Proportion of immunoreactivity. While healthy controls (Co) and PDAC patients show the lowest proportions of immunoreactivity, no statistically significant difference between non-invasive (low- and high-grade) IPMN can be detected. IPMN with associated cancer (IPMN-CA) show the highest percentage of immunoreactivity. Statistical significance is computed based on Kruskal-Wallis test with Dunn’s post hoc test. The boxplot depicts boxes whose upper and lower hinges correspond to the 25th and 75th percentiles. Whiskers cover the range from lowest or highest observed value. Significance is superimposed where *** or * correspond to *p*-values < 0.001 or 0.05, respectively.

Differences between groups were detected in the proportion of immunoreactive proteins per patient sample. While healthy controls showed a low median reactivity of approximately 0.4%, the low- and high-grade IPMN doubled that score (0.8% each). The highest rate of immunoreactivity was detected in IPMN with associated invasive cancer (1.8%). Interestingly, the immunoreactivity scores of PDAC patients were not statistically significantly different from that of healthy controls. The distributions of the proportion of immunoreactive proteins per patient sample within all patient groups are shown in Figure 1.

Adjustments for age left a total of 318 patient samples that were considered in the data analysis. Age and sex distribution of the remaining samples is shown in Table S2. The biomarker selection analysis was based on 168 proteins, which were seropositive for at least one patient and whose immunoreactivity pattern across samples was unique. A protein that was seropositive for all sera of one patient group could not be identified.

The multinomial lasso approach selected in total 14 proteins contributing to discrimination between diseases: ANXA4, CCKBR, CD99L2, CFI, FXYD7, GPR3, GPR173, HCFC1R1, HDAC3, PRDX2, RPL22, SLC22A15, TOR1B, and TP53. Model coefficients are provided in Table S3.

**Figure 2:**
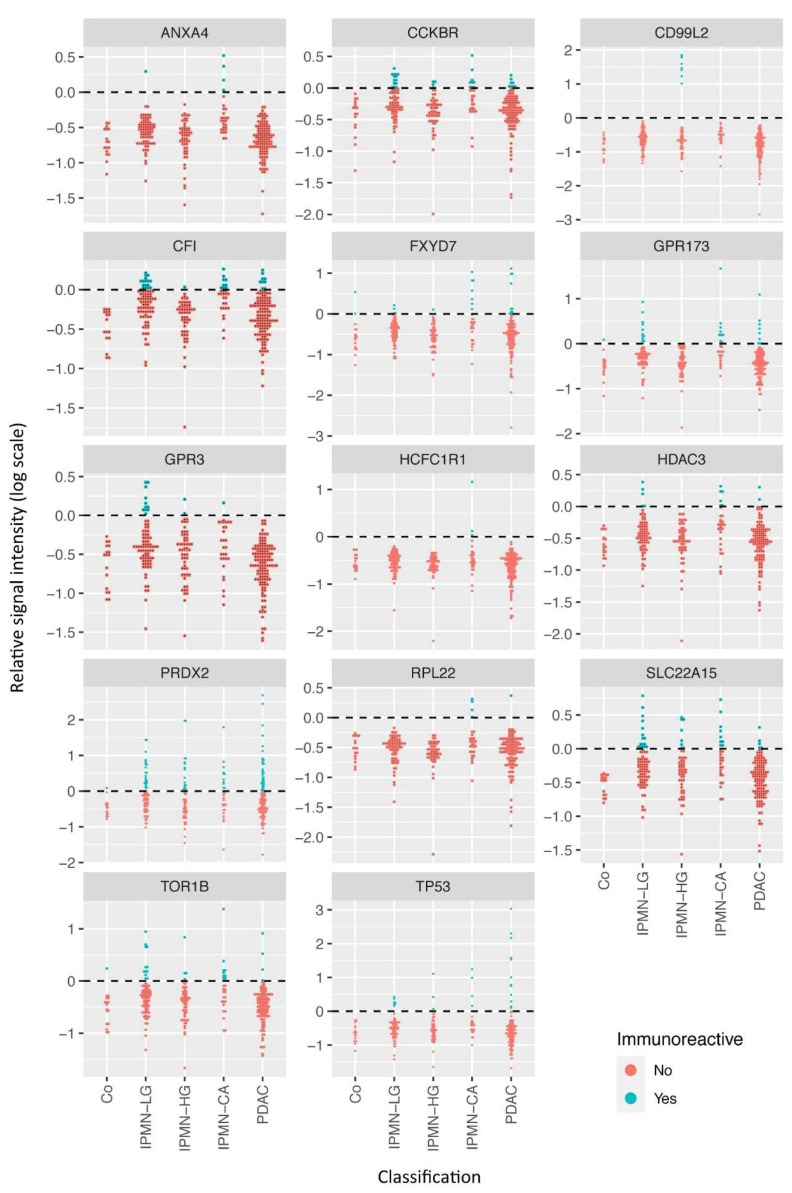
Relative signal intensities of the proteins selected for disease discrimination. The MFI values for the selected autoreactive antigens are shown after division by the thresholding value for positivity and on a logarithmic scale. Values above 0 (blue) are considered indicators of immunoreactivity.

To gain deeper insights into the immunoreactivity of the 14 proteins among different patient groups, their MFI divided by the threshold value stated as relative signal intensity are shown in Figure 2. 

**Figure 3:**
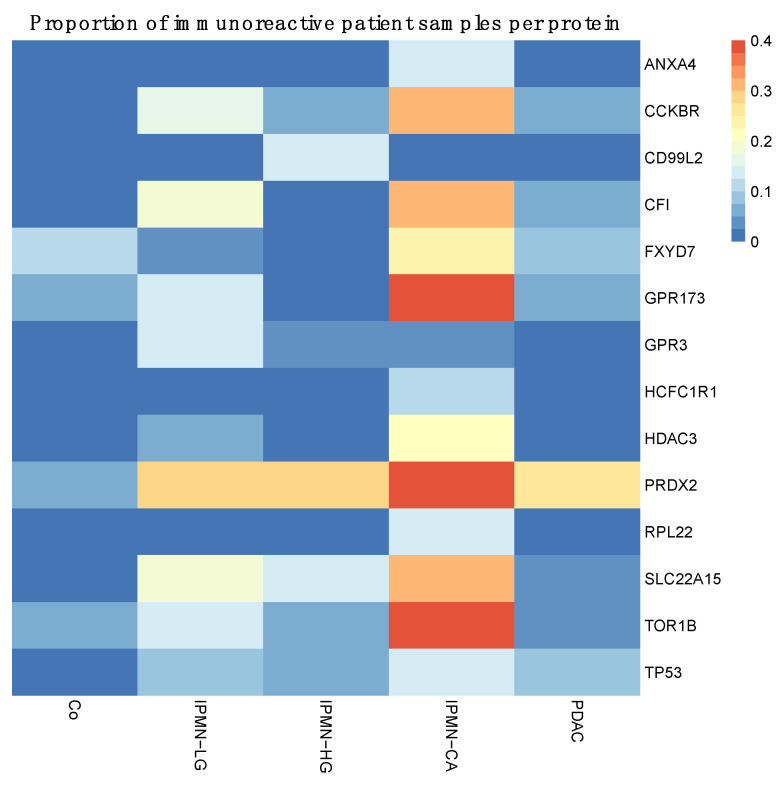
Heatmap of the proteins selected for disease discrimination and their proportion of reactivity among patients belonging to each disease class. Proteins are listed to the right of the heatmap. Each column represents a patient group.

The proportion of immunoreactive patient samples for each disease category (Co, IPMN-LG, IPMN-HG, IPMN-CA, and PDAC) and for each of the selected proteins is shown in a heatmap (Figure 3).

All disease classes (Co, IPMN-LG, IPMN-HG, IPMN-CA, and PDAC) were subjected to a pairwise comparison, yielding AUC- and M-values (Table S4). Low sensitivity of the autoantibodies leads to rather low AUC values. In consequence, the different disease stages could only be discriminated with rather low accuracy. This would make screening of patients without additional information about pancreatic lesions ineffective. Still, high specificities could make grade discrimination feasible for patients with radiological findings of cystic pancreatic lesions. For this reason, markers with high specificities were further explored for potential discrimination between IPMN grades.

This is of high clinical importance, as clinical care differs within IPMN grades. For patients with a low risk of developing an invasive carcinoma, surgery is not (yet) recommended, whereas IPMN with high-grade dysplasia or IPMN with associated invasive carcinoma are recommended for surgical intervention, if applicable. The ability to accurately discriminate between these two subgroups would therefore improve patient care. For this, high-grade (IPMN-HG) and invasive IPMN (IPMN-CA) cases were merged into the high-risk group (IPMN-HR). Low-risk IPMN (IPMN-LR) is equivalent to the IPMN-LG group. The previously identified 14 individual markers show high specificity of over 80% for discriminating high-risk from low-risk IPMN, except for PRDX2 (Table 2). One half of the molecules exhibited specificity values even above 90%. However, all marker proteins were clearly lacking sensitivity, which ranged from 3.5% to 31.4%. The average sensitivity across the 14 antigens was 12.9%.

A combination of marker antigens could enhance diagnostic robustness and accuracy if the individual marker molecules exhibit reasonable specificities. Thereby, a better sensitivity could be provided, because cases would be detected, which would be missed by a single marker. Of the 96 patients with IPMN-HR, 33 (34.4%) were seropositive for at least 1 of the 7 markers with a specificity above 90%. An autoantibody panel including those seven markers (CD99L2, RPL22, HCFC1R1, ANXA4, FXYD7, HDAC3, TP53) could add valuable information about the IPMN grade for approximately one third of patients, whereas the multinomial lasso model could not find a biomarker panel of reasonable power to distinguish between IPMN-HR and IPMN-LR.

Analysis of the gene ontology showed that 40% of the genes specific for IPMN-HR are linked to the negative regulation of the apoptotic process (*p* = 0.00098), namely ANXA4, HDAC3, TP53, and PRDX2. This could suggest that changes in the proper function of programmed cell death, which usually malfunctions in malignant cells, are more often present in high-risk IPMN.

KEGG pathway analysis identified HDAC3 and TP53 to be associated with the pathway of viral carcinogenesis. As the etiology of IPMN still remains uncertain, this could be a hint towards a viral infection being involved in the development of IPMN. 

## 4. Discussion

Because autoantibodies against tumor-associated proteins are produced during the early stages of tumorigenesis, they are ideal as biomarkers [38]. Most antibody profiling investigations determined antigens recognized by autoreactive antibodies to identify biomarkers for cancer [47,48,49]. Multiple reports have identified tumor-associated autoantibodies in multiple cancers, including colorectal cancer [50], bladder cancer [51], ovarian cancer, and pancreatic cancer [18,52]. However, a thorough data review reported that autoantibodies as biomarkers have an overall low sensitivity of 14%, although a high specificity of 95% [26]. Consequently, the diagnostic performance was lower compared to other types of markers in the serum [52].

Currently, the most prominent and widely used clinical marker for pancreatic tumors, including IPMN, is CA19-9. Elevated CA19-9 blood levels (>37 U/mL) in IPMN are associated with the presence of high-grade dysplasia and invasive carcinoma and could be used to differentiate between IPMN low- and IPMN high-risk individuals [53,54]. However, recent analyses using sera from a similar cohort as in the current study revealed that CA-19-9 performed poorly for stratifying IPMN low- and high-risk with an AUC of 0.545 [55]. A recent small but promising study identified MUC5AC in circulating extracellular vesicles as a possible predictor for malignancy in present IPMN, with a sensitivity of 82% and specificity of 100% [56].

Here, we explored the utility of autoreactive antibodies in the blood of patients with IPMN of various pathological grades (low-grade, high-grade dysplasia) and IPMN with associated invasive carcinoma. We compared the autoantibody patterns with that of samples collected from patients with early-stage PDAC (T1 and T2) and healthy blood donors and adjusted for age differences. In general, we observed a relatively low proportion of immunoreactive proteins, in accordance with earlier studies [27,57,58]. Specifically, the low mean reactivity of healthy individuals and PDAC patients was only 0.4%, which has been previously reported [18,20]. Notably, samples from patients with IPMN with associated carcinoma were the most seropositive, which is surprising as one would expect a kind of continuously developing pattern from high-risk IPMN to PDAC. The difference could indicate a development path towards a highly malign tumor that is different to PDAC, although pathologically relatively similar.

As for now, the etiology of IPMN remains mostly unclear [59]. Associations with diabetes mellitus, chronic pancreatitis, and family history of PDAC, which overlap with risk factors for PDAC itself, have been described [60]. Interestingly, the KEGG pathway analysis links the antigens identified in this study with viral carcinogenesis, which could be another possible explanation for the development of IPMN and other pancreatic lesions. The gene ontology analysis resulted in an association with the anti-apoptotic pathway, supporting the loss of self-regulatory cell functions during the progress toward malignancy.

Some experimental features of our study setup could have affected the overall performance. The study was designed retrospectively and performed with samples from a single high-volume center. The genes used for antigen synthesis were preselected, and not all could be studied because they were not available in the cDNA library. Moreover, the antigens were recombinant proteins expressed in a bacterial expression system devoid of posttranslational modification machinery, which might influence antibody binding. In addition, we applied a highly stringent signal detection by utilizing a threshold of five standard deviations of the background signal. Last, binary information—binding or no binding—was used for data analysis rather than considering signal intensities. Once these technical limitations were resolved, better results could be expected.

Despite the limitations, the abundance of antibodies against 14 proteins in patients’ sera was found to indicate advancing malignant progression of IPMN. The antigens TOR1B and GPR173 had already been described as markers for discriminating PDAC from chronic and autoimmune pancreatitis [20]. While diagnostic power is limited by the relatively low sensitivity, very high specificities were observed. By combining marker proteins, a reasonable sensitivity could be achieved, adding data about the IPMN status for about one third of the patients. Because the detection of IPMN with high-grade dysplasia and invasive carcinoma is often challenging and cross-imaging is frequently inadequate, a combination of diagnostic tools including radiology, serum analyses, and cytological/histological approaches could provide an accurate and robust approach for better decision-making for potential surgical intervention.

## 5. Conclusions

Considering the hitherto limited number of clinically routinely available biomarkers to distinguish IPMN of different grades, the integration of the autoreactive-antibody panel into current disease indicators may aid in the risk stratification of patients with IPMN for optimizing clinical management.

## Figures and Tables

**Table 1:** Clinicopathological parameters of IPMN and PDAC patients.

Parameter	Healthy Controls	IPMN-LG	IPMN-HG	IPMN-CA	PDAC
Samples (*n*)	54	91	66	30	137
Age (y)					
Mean ± SD	43.7 ± 15.7	63.9 ± 10.9	63.6 ± 10.2	66.9 ± 7.9	67.3 ± 10.5
Range	15–85	35–79	31–81	45–78	23–90
Median	41.0	65.4	64.9	67.6	68.7
Sex					
Male/Female	23/31	40/51	40/26	20/10	61/76
Location					
Head	-	54	35	16	104
Body	-	6	3	-	14
Tail	-	7	10	3	17
Multiple	-	19	15	11	-
*n*/a	-	5	3	-	2
AJCC/UICC stage 8th ed					
IA	-	-	-	12	15
IB	-	-	-	2	18
IIA	-	-	-	9	-
IIB	-	-	-	5	43
III	-	-	-	-	55
IV	-	-	-	2	6
Grading					
G1	-	-	-	4	1
G2	-	-	-	22	80
G3	-	-	-	4	53
*n*/a	-	-	-	-	3
Staging					
pT1	-	-	-	12	24
pT2	-	-	-	2	113
pT3	-	-	-	16	-
Lymph node					
N0	-	-	-	23	33
N1	-	-	-	7	44
N2	-	-	-	-	60
Metastasis					
M0	-	-	-	28	131
M1	-	-	-	2	6
R-classification					
R0	-	-	-	24	44
R0 (CRM+)	-	-	-	-	43
R1	-	-	-	6	47
Rx	-	-	-	-	1
*n*/a	-	-	-	-	2

**Table 2:** Calculation of specificity and sensitivity for the 14 marker antigens for making a distinction between IPMN-LR and IPMN-HR. On the left, markers with high specificity for IPMN-LR. On the right, markers with high specificity for IPMN-HR are listed. Specificity values above 90% are highlighted in bold writing.

	IPMN-LR			IPMN-HR	
Antigen	Sensitivity	Specificity	Antigen	Sensitivity	Specificity
GPR3	13.3%	**96.5%**	CD99L2	9.3%	**100.0%**
CFI	19.3%	88.4%	RPL22	4.7%	**100.0%**
GPR173	14.5%	87.2%	HCFC1R1	3.5%	**100.0%**
CCKBR	16.9%	84.9%	ANXA4	4.7%	**98.8%**
			FXYD7	9.3%	**95.2%**
			HDAC3	7.0%	**94.0%**
			TP53	9.3%	**91.6%**
			TOR1B	17.4%	85.5%
			SLC22A15	19.8%	80.7%
			PRDX2	31.4%	71.1%

## Data Availability

The data that support the findings of this study are available from the corresponding author upon reasonable request.

## Supplementary Materials

The following are available online at https://www.mdpi.com/article/10.3390/cancers14153562/s1, Supplementary Materials containing Table S1: cDNAs used for producing the protein microarray; Table S2: Cohort characteristics of the patients retained for the development of the classification models; Table S3: Multinomial lasso model fit: selected proteins and coefficients; Table S4: Apparent and cross-validated AUC values for discriminating different pancreatic malignancies.
