# Supplementary material for: Use of Autoreactive Antibodies in Blood of Patients with Pancreatic Intraductal Papillary Mucinous Neoplasms (IPMN) for Grade Distinction and Detection of Malignancy
Source: Cancers (Basel). 2022 Jul 22;14(15):3562. doi: 10.3390/cancers14153562 (PMC9332220; doi:10.3390/cancers14153562)
Supplement: Supplementary file 1 [file cancers-14-03562-s001.zip › cancers-1814524-supplementary.pdf]

Supplementary Material

Use of autoreactive antibodies in blood of patients with pancreatic intraductal papillary mucinous neoplasms (IPMN) for grade distinction and detection of malignancy

Table S1. cDNAs used for producing the protein microarray.

.	Gene symbol	Coding antigen	ORF length (bp)	PCR Type*
1	ACAD9	Acyl-Coa Dehydrogenase Family Member 9	2066	Taq-Taq
2	ACTA2	Actin Alpha 2	1334	Taq-Taq
3	ACTB	Actin Beta	1328	Taq-Taq
4	ACTG1	Actin Gamma 1	1328	Taq-Taq
5	ACTR3	Actin Related Protein 3	1457	Taq-Taq
6	ADA	Adenosine Deaminase	1292	Taq-Taq
7	ADAM32	Adam Metallopeptidase Domain 32	2564	Taq-LR
8	AFG3L1	Afg3 Atpase Family Gene 3-Like 1	569	Taq-Taq
9	AGT	Angiotensinogen	1658	Q5-Taq
10	AHSG	Alpha 2-Hs Glycoprotein	1304	Taq-Taq
11	AIRE	Autoimmune Regulator	1247	Taq-Taq
12	AKR1B1	Aldo-Keto Reductase Family 1, Member B1	1151	Taq-Taq
13	ALDH1A1	Retinal Dehydrogenase 1	1706	Taq-Taq
14	ANP32A	Acidic Nuclear Phosphoprotein 32 Family, Member A	917	Q5-Taq
15	ANXA1	Annexin A1	1241	Taq-Taq
16	ANXA11	Annexin A11	1718	Q5-Taq
17	ANXA2	Annexin A2	1220	Taq-Taq
18	ANXA4	Annexin A4	1166	Taq-Taq
19	AP2B1	Adaptor Related Protein Complex 2 Subunit Beta 1	3056	Taq-LR
20	APBB1	Amyloid Beta Precursor Protein Binding Family B Member 1	2327	Taq-LR
21	APOD	Apolipoprotein D	770	Taq-Taq
22	APOH	Apolipoprotein H	1238	Taq-Taq
23	ARFIP2	Adp-Ribosylationfactor Interacting Protein 2	1226	Taq-Taq
24	ARHGEF10	Rho Guanine Nucleotide Exchange Factor 10	1343	Taq-Taq
25	AVP	Arginine Vasopressin	695	Q5-Taq
26	BDNF	Brain-Derived Neurotrophic Factor	944	Taq-Taq
27	BIRC5	Baculoviral Iap Repeat Containing 5	629	Taq-Taq
28	BTD	Biotinidase	1832	Taq-Taq
29	C12orf48	Parp-1 Binding Protein (Parpbp)	1085	Q5-Taq
30	C4BPB	Complement Component 4 Binding Protein Beta	956	Taq-Taq
31	C6orf141	Chromosome 6 Open Reading Frame 141	914	Q5-Taq
32	C7	Complement C7	2732	Taq-LR
33	CA11	Carbonic Anhydrase 11	1187	Taq-Taq
34	CA12	Carbonic Anhydrase 12	626	Taq-Taq
35	CA8	Carbonic Anhydrase 8	662	Taq-Taq
36	CALML3	Calmodulin Like 3	650	Taq-Taq
37	CALR	Calreticulin	1454	Taq-Taq
38	CAMK4	Calcium/Calmodulin-Dependent Protein Kinase IV	437	Q5-Taq
39	CAPZB	Capping Actin Protein Of Muscle Z-Line Subunit Beta	1019	Taq-Taq
40	CCKBR	Cholecystokinin B Receptor	1544	Taq-Taq

41	CD24	CD24 Molecule	443	Taq-Taq
42	CD40	CD40 Molecule, TNF Receptor Superfamily Member 5	1034	Taq-Taq
43	CD79B	CD79B Molecule, Immunoglobulin-Associated Beta	440	Taq-Taq
44	CD99L2	CD99 Molecule Like 2	989	Taq-Taq
45	CDK4	Cyclin-Dependent Kinase 4	1112	Taq-Taq
46	CDKN2A	Cyclin Dependent Kinase Inhibitor 2A	518	Taq-Taq
47	CEACAM1	CEA Cell Adhesion Molecule 1	1607	Taq-Taq
48	CELA1	Chymotrypsin Like Elastase 1	977	Taq-Taq
49	CFH	Complement Factor H	1550	Taq-Taq
50	CFI	Complement Factor I	1334	Taq-Taq
51	CFL1	Cofilin-1	701	Taq-Taq
52	CIB2	Calcium and Integrin Binding Family Member 2	764	Taq-Taq
53	CLU	Clusterin	1550	Taq-Taq
54	CORO2B	Coronin (Actin Binding Protein) 2B	1628	Taq-Taq
55	CPB2	Carboxypeptidase B2	1472	Taq-Taq
56	CPN1	Carboxypeptidase N Subunit 1	1577	Taq-Taq
57	CRYBB1	Crystallin Beta B1	959	Taq-Taq
58	CTDSP1	CTD Small Phosphatase 1	986	Taq-Taq
59	CTNNBL1	Catenin Beta Like 1	1136	Taq-Taq
60	CTRB1	Chymotrypsinogen B	992	Taq-Taq
61	CYP3A5	Cytochrome P450 Family 3 Subfamily A Member 5	1709	Taq-Taq
62	DCTN1	Dynactin 1	797	Taq-Taq
63	DDAH1	Dimethylarginine Dimethylaminohydrolase 1	1058	Q5-Taq
64	DEFA1	Defensin Alpha 1	485	Taq-Taq
65	DNAJB1	DnaJ Heat Shock Protein Family (Hsp40) Member B1	1223	Taq-Taq
66	DTYMK	Deoxythymidylate Kinase	839	Taq-Taq
67	DYNC1I2	Dynein Cytoplasmic 1 Intermediate Chain 2	2039	Taq-Taq
68	EDARADD	Alpha Enolase Pseudogene	1367	Taq-Taq
69	EDN3	Endothelin 3	917	Taq-Taq
70	EIF1AD	Eukaryotic Translation Initiation Factor 1A Domain Containing	698	Taq-Taq
71	EIF1AY	Eukaryotic Translation Initiation Factor 1A Y-Linked	635	Taq-Taq
72	EIF1B	Eukaryotic Translation Initiation Factor 1B	542	Taq-Taq
73	EIF2S2	Eukaryotic Translation Initiation Factor 2, Subunit 2 Beta	1202	Taq-Taq
74	EIF3F	Eukaryotic Translation Initiation Factor 3, Subunit F	1274	Taq-Taq
75	EIF3G	Eukaryotic Translation Initiation Factor 3 Subunit G	1163	Taq-Taq
76	EIF4A3	Eukaryotic Translation Initiation Factor 4A3	1436	Taq-Taq
77	ENTPD7	Ectonucleoside Triphosphate Diphosphohydrolase 7	2015	Taq-LR
78	EPHX2	Epoxide Hydrolase 2	1868	Taq-Taq
79	EXT1	1 Exostosin Glycosyltransferase 1	2441	Taq-LR
80	EZR	Ezrin	1961	Taq-Taq
81	FAM13A	Family with Sequence Similarity13 Member A	809	Taq-Taq
82	FCGR2B	Fc Fragment of IgG Receptor IIb	1133	Taq-Taq
83	FDPS	Farnesyl Diphosphate Synthase	1460	Taq-Taq
84	FH	Fumarate Hydratase	1733	Taq-Taq
85	FMO2	Flavin Containing Monooxygenase 2	1616	Taq-Taq
86	FSTL1	Follistatin Like 1	1127	Taq-Taq
87	FXYD7	Fxyd Domain Containing Ion Transport Regulator 7	443	Taq-Taq
88	G6PD	Glucose-6-Phosphate 1-Dehydrogenase	1748	Taq-Taq
89	GABARAPL2	Gaba Type A Receptor Associated Protein Like 2	554	Taq-Taq
90	GAD1	Glutamate Decarboxylase 1	875	Taq-Taq
91	GADD45A	Growth Arrest and DNA Damage Inducible Alpha	698	Q5-Taq

92	GAPDH	Glyceraldehyde-3-Phosphate Dehydrogenase	1208	Taq-Taq
93	GAS2	Growth Arrest-Specific 2	626	Taq-Taq
94	GLUD2	Glutamate Dehydrogenase 2	995	Taq-Taq
95	GNAS	GNAS Complex Locus	1388	Taq-Taq
96	GNB2L1	Guanine Nucleotide Binding Protein (G Protein), Beta Polypeptide 2-Like	1154	Taq-Taq
97	GNL1	G Protein Nucleolar 1 (Putative)	2024	Taq-Taq
98	GPR173	G Protein-Coupled Receptor 173	1322	Taq-Taq
99	GPR3	G Protein-Coupled Receptor 3	1193	Taq-Taq
100	GSTP1	Glutathione S-Transferase Pi 1	833	Taq-Taq
101	GTPBP8	GTP-Binding Protein 8 (Putative)	1055	Taq-Taq
102	HAUS8	Haus Augmin Like Complex Subunit 8	947	Taq-Taq
103	HAX1	HCLS1 Associated Protein X-1	1040	Taq-Taq
104	HBB	Hemoglobin Subunit Beta	644	Taq-Taq
105	HCFC1R1	Host Cell Factor C1 Regulator 1	560	Taq-Taq
106	HDAC3	Histone Deacetylase 3	1487	Taq-Taq
107	HERPUD1	Homocysteine Induc. ER Protein with Ubiquitin Like Domain 1	1376	Taq-Taq
108	HIBCH	3-Hydroxyisobutyryl-Coa Hydrolase	1202	Taq-Taq
109	HIST2H2BE	Histone H2B	581	Taq-Taq
110	HM13	Histocompatibility Minor 13	1334	Q5-Taq
111	HNRNPA2B1	Heterogeneous Nuclear Ribonucleoprotein A2	950	Taq-Taq
112	HNRPLL	Heterogeneous Nuclear Ribonucleoprotein L Like	1028	Taq-Taq
113	HPX	Hemopexin Precursor	965	Taq-Taq
114	HYAL2	Hyaluronidase 2	1622	Taq-Taq
115	ICAM1	Intercellular Adhesion Molecule 1	1799	Taq-Taq
116	IDH3A	Isocitrate Dehydrogenase 3 Alpha	1301	Taq-Taq
117	IER2	Immediate Early Response 2	872	Q5-Taq
118	IFITM3	Interferon-Induced Transmembrane Protein 3	602	Taq-Taq
119	IGF2BP3	Kh-Domain Containing Protein Over Expressed In Cancer	1940	Taq-Taq
120	IGFBP1	Insulin Like Growth Factor Binding Protein 1	980	Q5-Taq
121	IGFBP5	Insulin Like Growth Factor Binding Protein 5	1019	Q5-Taq
122	IL1A	Interleukin 1, Alpha	1016	Taq-Taq
123	IMMP1L	Inner Mitochondrial Membrane Peptidase Subunit 1	701	Q5-Taq
124	INS	Insulin	533	Taq-Taq
125	ITIH5	Inter-Alpha-Trypsin Inhibitor Heavy Chain Family Member 5	2387	Taq-LR
126	KEL	Kell Metallo-Endopeptidase	2399	Taq-LR
127	KIF22	Kinesin Family Member 22	2198	Taq-Taq
128	KLF6	Kruppel-Like Factor 6	983	Taq-Taq
129	KLHL2	Kelch Like Family Member 2	1982	Taq-Taq
130	KLK10	Kallikrein-Related Peptidase 10	1031	Q5-Taq
131	KLKB1	Kallikrein B1	2117	Taq-LR
132	KRT6A	Keratin 6A	1895	Taq-Taq
133	KRT8	Keratin 8	1040	Taq-Taq
134	KRTAP4-12	Keratin Associated Protein 4-12	806	Taq-Taq
135	LDHA	Ldh A Chain Isoform 1	1199	Taq-Taq
136	LDHB	Lactate Dehydrogenase B	1205	Taq-Taq
137	LENG1	Leukocyte Receptor Cluster Member 1	995	Taq-Taq
138	LRG1	Leucine Rich Alpha-2-Glycoprotein 1	1244	Taq-Taq
139	LRRC49	Leucine Rich Repeat Containing 49	2258	Taq-Taq
140	LTF	Lactotransferrin	2336	Taq-LR
141	LUC7L	LUC7 Like	1178	Taq-Taq

142	MAPK9	Mitogen-Activated Protein Kinase 9	1475	Taq-Taq
143	MAPRE3	Microtubule Associated Protein RP/EB Family Member 3	1046	Taq-Taq
144	MC3R	Melanocortin 3 Receptor	1283	Taq-Taq
145	MDH1	Malate Dehydrogenase	1205	Taq-Taq
146	MED1	Mediator Complex Subunit 1	1871	Taq-Taq
147	MEST	Mesoderm Specific Transcript	1208	Q5-Taq
148	MFN1	Mitofusin 1	2426	Taq-Taq
149	MIA	MIA SH3 Domain Containing	596	Taq-Taq
150	MRPL12	Mitochondrial Ribosome Protein L12	797	Taq-Taq
151	MSH2	Homo Sapiens Muts Homolog 2	3005	Taq-LR
152	Muc1	Mucin 1	995	Taq-Taq
153	NR2E3	Nuclear Receptor Subfamily 2- Group E- Member 3	1169	Q5-Taq
154	NUP62	Nucleoporin 62	1769	Taq-Taq
155	P2RY6	Pyrimidinergic Receptor P2Y (G-Protein Coupled, 6)	1187	Q5-Taq
156	P4HB	Prolyl 4-Hydroxylase Subunit Beta	1727	Taq-Taq
157	PAICS	Phosphoribosylaminoimidazole Carboxylase and Phosphoribosyl-aminoimidazolesuccinocarboxamide Synthase	1478	Taq-Taq
158	PARP3	Poly (ADP-Ribose) Polymerase 3	1802	Taq-Taq
159	PCNA	Proliferating Cell Nuclear Antigen	986	Taq-Taq
160	PDLIM1	PDZ And LIM Domain 1	1190	Taq-Taq
161	PENK	Proenkephalin	1004	Taq-Taq
162	PEPD	Peptidase D	1682	Taq-Taq
163	PGK1	Phosphoglyceratekinase 1	1454	Q5-Taq
164	PKM2	Pyruvate Kinase M1/2	1796	Taq-Taq
165	PLEKHB2	Pleckstrin Homology Domain Containing B2	869	Taq-Taq
166	PNLIPRP2	Pancreas Lipase-Related Protein 2	1610	Q5-Taq
167	PPARG	Peroxisome Proliferative Activated Receptor Gamma	1634	Taq-Taq
168	PPBP	Pro-Platelet Basic Protein	587	Taq-Taq
169	PPY	Pancreatic Polypeptide	488	Taq-Taq
170	PRDX2	Peroxiredoxin 2	629	Taq-Taq
171	PRDX4	Peroxiredoxin-4	1016	Taq-Taq
172	PRMT6	Protein Arginine Methyltransferase 6	1151	Q5-Taq
173	PRSS2	Protease, Serine, 2 (Trypsin2)	920	Taq-Taq
174	PSAT1	Phosphoserine Aminotransferase 1	1313	Taq-Taq
175	PSCA	Prostate Stem Cell Antigen	572	Taq-Taq
176	PSMA1	Proteasome Subunit Alpha Type-1 Isoform 2	992	Taq-Taq
177	PSMC4	Proteasome 26S Subunit, Atpase 4	1457	Taq-Taq
178	PSME3	Proteasome Activator Subunit 3	965	Taq-Taq
179	PTENP1	Phosphatase and Tensin Homolog Pseudogene 1	1310	Taq-Taq
180	PTGES3	Prostaglandin E Synthase 3	683	Taq-Taq
181	PTPRA	Protein Tyrosine Phosphatase Receptor Type A	2582	Taq-LR
182	Rad51	Rad51 Recombinase	929	Taq-Taq
183	RAD51C	Rad51 Paralog C	605	Taq-Taq
184	RAP1GDS1	Rap1, GTP-GDP Dissociation Stimulator 1	2027	Taq-LR
185	RARA	Retinoic Acid Receptor, Alpha	1589	Q5-Taq
186	RBBP8	Retinoblastoma Binding Protein 8	2909	Taq-LR
187	REG1A	Regenerating Family Member 1 Alpha	701	Taq-Taq
188	REG1B	Regenerating Family Member 1 Beta	701	Taq-Taq
189	REG4	Regenerating Family Member 4	677	Taq-Taq
190	RIT2	Ras Like Without Caax 2	854	Taq-Taq
191	RNF10	Ring Finger Protein 10	2636	Taq-LR

192	RNF138	Ring Finger Protein 138	938	Q5-Taq
193	RPL13	Ribosomal Protein L13	836	Q5-Taq
194	RPL22	Ribosomal Protein L22	587	Taq-Taq
195	RPL4	Ribosomal Protein L4	1484	Taq-Taq
196	RPSA	Laminin-Binding Protein Galectin 3/Ribosomal Protein Sa	1088	Taq-Taq
197	RRP8	Ribosomal RNA Processing 8	1571	Taq-Taq
198	RUNX1	Runt Related Transcription Factor 1	1643	Q5-Taq
199	S100A4	S100 Calcium Binding Protein A4	506	Taq-Taq
200	S100A6	S100 Calcium Binding Protein A6	473	Taq-Taq
201	SEC13	SEC13 Homolog, Nuclear Pore and COPII Coat Complex Component	1169	Taq-Taq
202	SERPINA10	Serpin Family A Member 10	1535	Taq-Taq
203	SERPINC1	Serpin Family C Member 1	980	Taq-Taq
204	SERPINE2	Serpin Family E Member 2	1397	Taq-Taq
205	SHOC2	Shoc2 Leucine Rich Repeat Scaffold Protein	1949	Taq-Taq
206	SLC22A15	Solute Carrier Family 22 Member 15	1808	Taq-Taq
207	SLC30A8	Solute Carrier Family 30 Member 8	1163	Q5-Taq
208	SMAD2	Smad Family Member 2	1604	Taq-Taq
209	SMAD4	Smad Family Member 4	1859	Taq-Taq
210	SMAD5	Smad Family Member 5	1598	Taq-Taq
211	SMAD9	Smad Family Member 9	1493	Taq-Taq
212	SMOX	Spermine Oxidase	1709	Taq-Taq
213	SOD2	Superoxide Dismutase 2 (Mitochondrial)	623	Taq-Taq
214	SOX8	SRY-Box Transcription Factor 8	1541	Q5-Taq
215	SPARC	Secreted Protein Acidic and Cysteine Rich	1112	Taq-Taq
216	SPATA1	Spermatogenesis Associated 1	863	Taq-Taq
217	SPINK1	Serine Peptidase Inhibitor, Kazal Type 1	440	Taq-Taq
218	SPTLC2	Serine Palmitoyltransferase, Long Chain Base Subunit 2	1889	Q5-Taq
219	SQSTM1	Sequestosome 1	1271	Taq-Taq
220	SRGAP1	Slit-Robo Rho Gtpase Activating Protein 1	1640	Taq-Taq
221	STK33	Serine/Threonine Kinase 33	1745	Taq-Taq
222	STMN3	Stathmin 3	743	Taq-Taq
223	SYCN	Syncollin	605	Q5-Taq
224	TAGLN	Transgelin	806	Taq-Taq
225	TALDO1	Transaldolase	1157	Taq-Taq
226	TARBP2	Tarbp2 Subunit of Risc Loading Complex	947	Taq-Taq
227	TGM2	Transglutaminase 2	1847	Taq-Taq
228	TIMM44	Translocase of Inner Mitochondrial Membrane 44	1559	Taq-Taq
229	TIMP2	Timp Metallopeptidase Inhibitor 2	863	Q5-Taq
230	TMOD1	Tropomodulin 1	1280	Taq-Taq
231	TMSB10	Thymosin Beta 10	335	Taq-Taq
232	TNF	Tumor Necrosis Factor	902	Taq-Taq
233	TNP1	Transition Protein 1	368	Taq-Taq
234	TOR1B	Torsin Family 1 Member B	1211	Taq-Taq
235	TP53	Tumor Protein P53	1382	Taq-Taq
236	TPI1	Triosephosphateisomerase1	950	Taq-Taq
237	TUBA3E	Tubulin Alpha 3E	1553	Taq-Taq
238	TUFM	Elongation Factor Tu	1568	Taq-Taq
239	TXN2	Thioredoxin 2	701	Taq-Taq
240	UBR2	Ubiquitin Protein Ligase E3 Component N-Recognin 2	1520	Taq-Taq
241	ULK4	Unc-51 Like Kinase 4	1943	Taq-Taq

242	UROD	Uroporphyrinogen Decarboxylase	1304	Taq-Taq
243	UROS	Uroporphyrinogen III Synthase	998	Taq-Taq
244	VHL	Von Hippel-Lindau Tumor Suppressor	719	Q5-Taq
245	VIM	Vimentin	1601	Taq-Taq
246	WARS	Tryptophanyl-TRNA Synthetase 1	1616	Taq-Taq
247	WDR45	WD Repeat Domain 45	1283	Taq-Taq
248	WFDC2	Wap Four-Disulfide Core Domain 2	575	Taq-Taq
249	ZNF695	Zinc Finger Protein 695	719	Taq-Taq

*) First and second run of PCR; Taq: standard PCR with Taq-polymerase, LR: PCR with long-range polymerase, Q5: PCR with Q5 high fidelity polymerase.

Table S2. Cohort characteristics of the patients retained for the development of the classification models.

A: Sex.

Sex		n_{Co}	$\%_{Co}$	$n_{IPMN-LG}$	$\%_{IPMN-LG}$	$n_{IPMN-HG}$	$\%_{IPMN-HG}$	$n_{IPMN-CA}$	$\%_{IPMN-CA}$	n_{PDAC}	$\%_{PDAC}$	n_{All}	$\%_{All}$
	Females	10	58.8	47	56.6	23	40.4	10	34.5	73	55.3	163	51.3
	Males	7	41.2	36	43.4	34	59.6	19	65.5	59	44.7	155	48.7
$p = 0.08$	All	17	100.0	83	100.0	57	100.0	29	100.0	132	100.0	318	100.0

p= *p*-value.

B: Age distribution.

Age	Group	n	Min	q_1	Median	Mean	q_3	Max	SD	IQR
	Co	17	48.0	52.0	65.0	63.1	70.0	85.0	11.3	18.0
	IPMN-LG	83	46.2	60.1	67.8	66.0	73.0	79.8	8.5	12.9
	IPMN-HG	57	46.7	60.2	66.6	65.5	71.1	81.6	8.3	10.9
	IPMN-CA	29	45.8	61.2	67.6	66.7	73.1	78.2	8.1	11.8
	PDAC	132	47.4	61.0	69.2	68.2	76.3	90.0	9.5	15.2
$p = 0.09$	All	318	45.8	60.1	68.0	66.7	73.5	90.0	9.1	13.5

N = sample size, Min = minimum, q_1, q_3 = 1st, 3rd quartile (25th, 75th percentile), Max = maximum, SD = standard deviation, IQR = interquartile range.

Table S3. Multinomial lasso model fit: selected proteins and coefficients. Omitted coefficients are equal to 0. The penalization parameter is chosen to be 1 standard error away from the optimal cross-validation one. Each coefficient can be interpreted similarly to logistic regression for each pair of disease classes, i.e., for given disease classes A and B, the difference between the coefficients of class A and B for each parameter estimates the difference in the log-ratio of the probabilities to belong to class A and B for a unit change of the corresponding parameter (in the case of proteins, as the protein becomes immunoreactive). **(A)** Multinomial model with classes Co, IPMN-LG, IPMN-HG, IPMN-CA, and PDAC; **(B)** Multinomial model after merging of IPMN-HG and IPMN-CA to form the IPMN-HR class, IPMN-LR coincides with IPMN-LG.

(A).

Parameter	Co	IPMN-LG	IPMN-HG	IPMN-CA	PDAC
(Intercept)	2.679	-0.009	0.279	-1.381	-1.568
Age	-0.034	0.002	-0.007	0.012	0.027
Sex Male	-0.340	-0.263	0.427	0.388	-0.212
ANXA4				0.323	
CCKBR	-0.168			0.558	
CD99L2			1.809		
CFI	-0.283	0.303		0.600	
FXYD7	0.237			0.158	
GPR173			-0.652	0.215	

GPR3		1.015			
HCFC1R1				1.349	
HDAC3				0.195	
PRDX2	-0.899				
RPL22				0.301	
SLC22A15	-0.513				-0.107
TOR1B				0.508	-0.141
TP53	-0.245				

(B).

Parameter	Co	IPMN-LR	IPMN-HR	PDAC
(Intercept)	2.352	-0.215	-0.435	-1.702
Age	-0.031	0.004	0.001	0.027
Sex Male	-0.230	-0.182	0.541	-0.128
CD99L2			0.859	
CFI	-0.397	0.310		
FXYD7	0.252			
GPR3		0.732		
PRDX2	-0.905			
SLC22A15	-0.381		0.489	
TOR1B				-0.121
TP53	-0.133			

Table S4. Apparent and cross-validated AUC values for discriminating different pancreatic malignancies. (A) Multinomial model with classes Co, IPMN-LG, IPMN-HG, IPMN-CA, and PDAC; (B) Multinomial model after the merging of IPMN-HG and IPMN-CA to form the IPMN-HR class, IPMN-LR coincides with IPMN-LG.

Comparison	Apparent AUC	Cross-valid. AUC
A: Individual pairwise comparisons		
Overall M-value	0.73	0.62
Co vs. IPMN-LG	0.73	0.60
Co vs. IPMN-HG	0.75	0.65
Co vs. IPMN-CA	0.85	0.70
Co vs. PDAC	0.69	0.63
IPMN-LG vs. IPMN-HG	0.71	0.64
IPMN-LG vs. IPMN-CA	0.75	0.54
IPMN-LG vs. PDAC	0.59	0.53
IPMN-HG vs. IPMN-CA	0.81	0.67
IPMN-HG vs. PDAC	0.68	0.63
IPMN-CA vs. PDAC	0.77	0.61
B: Pairwise comparisons after merging of IPMN-HG and IPMN-CA		
Overall M-value	0.68	0.60
Co vs. IPMN-LR	0.75	0.61
Co vs. IPMN-HR	0.77	0.65
Co vs. PDAC	0.70	0.63
IPMN-LR vs. IPMN-HR	0.64	0.55
IPMN-LR vs. PDAC	0.59	0.53
PMN-HR vs. PDAC	0.66	0.60
